# COX-2 Expression in Paired Primary Tumors and Lymph Node Metastases: An Exploratory Clinicopathological Study

**DOI:** 10.3390/cancers18172740

**Published:** 2026-08-24

**Authors:** Kamil Kowalczyk, Bartosz Małkiewicz, Aleksandra Piotrowska, Paweł Kiełb, Krzysztof D. Dudek, Adam Gurwin, Jakub Karwacki, Dariusz Kowalczyk, Wojciech Krajewski, Tomasz Szydełko, Agnieszka Hałoń, Piotr Dzięgiel, Maciej Kaczorowski

**Affiliations:** 1 Department of Minimally Invasive and Robotic Urology, University Centre of Excellence in Urology, Wroclaw Medical University, 50-556 Wroclaw, Poland; bartosz.malkiewicz@umw.edu.pl (B.M.);; 2 Division of Histology and Embryology, Department of Human Morphology and Embryology, Wroclaw Medical University, 50-368 Wroclaw, Poland; 3 Military Institute of Medicine—National Research Institute, 4th Military Clinical Hospital in Wroclaw, 50-981 Wroclaw, Poland; krdudek@4wsk.pl; 4 Department of Anatomy, School of Medicine in Opole, University of Opole, 45-052 Opole, Poland; 5 Department of Clinical and Experimental Pathology, Wroclaw Medical University, 50-556 Wroclaw, Poland

**Keywords:** cyclooxygenase-2 (COX-2), prostate cancer, prognostic marker, immunohistochemistry, H-score, radical prostatectomy

## Abstract

Background/Objectives: Prostate cancer remains one of the most common malignancies in men, and prognosis remains particularly challenging in patients with lymph node metastases. Cyclooxygenase-2 (COX-2), an inducible enzyme involved in inflammation and tumor progression, has been investigated as a potential prognostic biomarker and therapeutic target, but its expression in lymph node metastases remains unclear. While COX-2 overexpression in primary prostate tumors has been reported, its expression in lymph node metastases has not been thoroughly investigated. Therefore, this study aimed to compare COX-2 expression in primary prostate tumors and corresponding lymph node metastases and to evaluate its association with clinicopathological characteristics and survival. Methods: This study included 77 treatment-naïve patients with prostate cancer and histologically confirmed lymph node metastases who underwent radical prostatectomy with extended lymphadenectomy. COX-2 expression was assessed using immunohistochemistry in paired samples from primary tumors and corresponding lymph node metastases. Statistical comparisons were conducted using the Mann–Whitney U test and survival analyses were performed using Kaplan–Meier curves with the log-rank test. Results: COX-2 expression was detected in both primary tumors and lymph node metastases, with no significant difference in staining intensity between the two sites. High COX-2 expression in primary tumors was significantly associated with a higher percentage of involved lymph nodes (30.0% vs. 11.8%, *p* = 0.026), elevated postoperative PSA levels (1.98 vs. 0.10 ng/mL, *p* = 0.007), and reduced surgical radicality (11.1% vs. 63.2%, *p* = 0.008). Moreover, elevated COX-2 expression in both primary and metastatic tissues correlated with worse five-year overall survival (41.7% vs. 92.8%, *p* = 0.033; and 40.0% vs. 95.6%, *p* < 0.001, respectively). Conclusions: High COX-2 expression is associated with adverse clinicopathological features and poorer survival in lymph node–positive prostate cancer. The association of COX-2 expression with the extent of lymph node involvement and survival suggests that COX-2 may have prognostic value in this setting. However, the present findings do not establish a causal role of COX-2 in disease progression or lymphangiogenesis. Further studies are warranted to validate the prognostic significance of COX-2 and to clarify its potential biological and therapeutic relevance in lymph node–positive prostate cancer.

## Simple Summary

Prostate cancer is one of the most common cancers among men, and the prognosis of patients with lymph node metastases remains difficult to predict. Cyclooxygenase-2, a protein involved in inflammation and tumor progression, may serve as a marker of aggressive disease. We analyzed clinical and pathological data from 77 patients with prostate cancer and lymph node metastases and evaluated cyclooxygenase-2 expression in primary prostate tumors and corresponding metastatic lymph nodes. Expression levels were similar in both tissues. High cyclooxygenase-2 expression in primary tumors was associated with a greater percentage of involved lymph nodes, higher postoperative prostate-specific antigen levels, and poorer overall survival. High expression in lymph node metastases was also associated with poorer survival. These findings suggest that cyclooxygenase-2 may have potential prognostic value in lymph node-positive prostate cancer; however, larger prospective studies are required to confirm its potential clinical application.

## 1. Introduction

Prostate cancer (PC) remains the second most common malignant tumor in men worldwide. Although the introduction of prostate-specific antigen (PSA) screening in the 1990s has significantly enhanced early detection, its diagnostic specificity is limited, as elevated PSA levels may also occur in benign conditions such as prostatitis and benign prostatic hyperplasia [1,2]. PSA testing also leads to PC overdiagnosis and, hence, its overall clinical benefit remains a subject of ongoing debate within the medical and scientific communities [3,4]. Consequently, there is a need for additional robust biomarkers that can guide risk stratification and therapy. To date, no such additional biomarker has been approved by urological guidelines for PC diagnosis.

The association between inflammation and cancer development is well established and continues to be a focus of oncological research. Chronic inflammation is estimated to contribute to nearly one-quarter of all malignancies [5,6]. Inflammatory cells found in the tumor microenvironment contribute to the neoplastic process by facilitating cell proliferation, survival and migration. This phenomenon has been investigated as a potential target for antitumor therapy using anti-inflammatory drugs [7]. One of the widely studied targets for potential therapy is cyclooxygenase-2 (COX-2)—an enzyme that catalyzes the conversion of prostaglandins (PGs) from arachidonic acid. In contrast to its constitutive isoform COX-1, expressed in most tissues and responsible for maintaining cellular homeostasis, COX-2 is an inducible enzyme upregulated by cytokines, growth factors and tumor promoters in pathological states including inflammation and cancers [8,9].

COX-2 overexpression was reported in several malignancies including gastric, liver, breast and colorectal cancer [10,11,12,13]. Beyond its role in prostaglandin synthesis, COX-2 is involved in multiple processes related to carcinogenesis. In tumor tissue, COX-2 co-localizes with vascular endothelial growth factor (VEGF) and transforming growth factor beta (TGF-β). A high expression of COX-2 and VEGF is closely associated with increased microvascular density, with new blood vessels proliferating in tumor regions exhibiting COX-2 expression [14]. Beyond its role in promoting angiogenesis, COX-2 overexpression enhances tumor cell invasiveness in vitro, likely mediated through CD44 upregulation [15]. Furthermore, cells with forced COX-2 expression attenuate the induction of apoptosis by non-steroidal anti-inflammatory drugs (NSAIDs) and 5-fluorouracil (5-FU), mediated by reduced membrane death receptor-5 (DR-5), cytochrome c and caspase activation, suggesting a role of COX-2 in apoptosis regulation [16,17]. Finally, COX-2-mediated prostaglandin signalling in proliferative compartments of epithelial tissues results in inhibition of cell differentiation, hyperplasia and dysplasia [18,19].

Previous studies have investigated COX-2 expression in primary prostate cancer and reported a correlation between COX-2 expression and key clinicopathological features, including Gleason score, tumor stage, lymph node (LN) metastases, and oncological outcomes. These studies underscore not only the role of inflammation in carcinogenesis, but also highlight the connection between COX-2 and VEGF-C levels, and consequently, lymphangiogenesis and LN metastases [20,21,22,23,24,25].

However, to the best of our knowledge, data specifically comparing COX-2 expression in paired primary prostate tumors and matched lymph node metastases remain limited. Data on COX-2 expression in lymph node metastases from other tumor types are also limited, despite reported associations between COX-2 and lymphangiogenesis [26,27,28]. It is therefore unclear to what extent COX-2 expression patterns are preserved between primary and nodal metastatic sites. PC patients with LN metastases constitute a clinically distinct subgroup at increased risk of disease progression. Although current European Association of Urology (EAU) Guidelines recommend several postoperative management strategies, including observation, androgen deprivation therapy, or combined radiotherapy and androgen deprivation therapy depending on disease characteristics, optimal treatment selection remains challenging [29]. Based on the established biological role of COX-2 in inflammation-driven tumor progression and metastatic dissemination, we hypothesized that COX-2 expression may be preserved in lymph node metastases and associated with adverse clinicopathological features and oncological outcomes. Therefore, we analyzed clinicopathological correlations of COX-2 immunoreactivity in primary PCs and matched LN obtained from lymph node-positive PC patients [26,27,28,29].

## 2. Methods

### 2.1. Patients and Clinicopathological Data

From an initial cohort of 95 patients, 77 men aged 49 to 79 years with PC with nodal metastases were included in the final analysis. Patients with incomplete clinical records or non-evaluable tumor tissue microarray (TMA) sections from either the primary tumor or LN metastasis, as well as patients with a history of another malignancy, were excluded from the analysis. Each patient underwent radical prostatectomy (RP) with extended pelvic lymphadenectomy at the University Urology Centre in Wrocław, Poland, between January 2012 and September 2018. All patients were clinically node-negative (cN0) at preoperative staging. LN metastases were identified only postoperatively by histopathological examination of the lymphadenectomy specimens, resulting in a pathologically node-positive (pN1) study cohort. RP was accompanied by bilateral extended pelvic lymph node dissection. The institutional dissection template included removal of lymphatic tissue from the external iliac and internal iliac regions, the obturator fossae, the common iliac regions cranially up to the ureteric crossing, and the presacral region. All patients were treatment-naïve and did not receive any form of antitumor therapy—including androgen deprivation therapy, chemotherapy, or radiotherapy—prior to radical prostatectomy. Tumor staging and grading were performed in accordance with the 8th edition of the American Joint Committee on Cancer (AJCC) staging and the 2019 International Society of Urological Pathology (ISUP) consensus on prostate cancer grading, respectively [30,31]. Histopathological evaluation was independently performed by two experienced genitourinary pathologists, and any diagnostic discrepancies were resolved through consensus during a joint review. Risk stratification for biochemical recurrence in localized and locally advanced prostate cancer was based on the guidelines of the European Association of Urology [29]. Postoperative serum PSA was measured for the first time approximately six weeks after radical prostatectomy. For the purposes of this study, surgical radicality was defined solely according to the biochemical criterion of a postoperative PSA level < 0.2 ng/mL at this assessment. Surgical margin status was evaluated histopathologically and analyzed separately and was not included in the definition of surgical radicality. Overall survival was calculated from the date of radical prostatectomy, regardless of the cause of death. The follow-up period ranged from 0 to 106 months. All clinical and histopathological background data were obtained from archived hospital records.

### 2.2. Tissue Samples and Immunohistochemistry

Archival formalin-fixed and paraffin-embedded tissue samples were used for COX-2 immunohistochemistry (IHC). For each patient, paired specimens from the primary tumor and corresponding LN metastasis were analyzed. TMAs had been constructed previously [32]. Briefly, for each patient, three representative cores containing viable PC tissue were selected from the primary tumor. For the analysis of lymph node metastases, three independent 1.5-mm tissue cores were obtained from a single metastatic LN for each patient. In patients with multiple metastatic LNs, the LN containing the largest metastatic deposit was selected for tissue microarray construction to maximize tissue representation. Representative tumor areas were identified on hematoxylin and eosin-stained sections by an experienced genitourinary pathologist, avoiding necrosis and extensive tissue artifacts. The final patient-level COX-2 expression score was calculated as the mean H-score of the three corresponding cores, which was intended to partially account for intralesional heterogeneity. IHC staining was performed on 4 μm TMA paraffin sections using the Autostainer Link48 system (Agilent, Santa Clara, CA, USA). Slides were deparaffined and epitope retrieval was conducted by treating the slides with EnVision FLEX Target Retrieval Solution (Agilent) (97 °C, 20 min; pH 9) using PTLink (Agilent). To block endogenous peroxidase activity, slides were incubated with EnVision FLEX Peroxidase-Blocking Reagent (5 min; Agilent). Subsequently, a rabbit monoclonal primary antibody against COX-2 (1:100, MA5-42821, Thermo Fisher, Cambridge, UK) was applied for 20 min. Reactions were visualized using 3,3’-diaminobenzidine (DAB) as the substrate for horseradish peroxidase, with a 10-min incubation. All sections were counterstained with EnVision FLEX Hematoxylin (Agilent) for 5 min. Finally, the slides were dehydrated in graded ethanol concentrations (70%, 96%, absolute) followed by xylene, and mounted with coverslips using Dako Mounting Medium (Agilent). All slides were scanned using the Pannoramic MIDI system (3DHistech, Budapest, Hungary) for digital evaluation. A semi-quantitative evaluation of IHC staining was performed using the H-score method. Staining intensity was graded on a scale from 0 (negative) to 3 (strong), and each intensity score was multiplied by the corresponding percentage of positively stained tumor cells. The final H-score, calculated as the sum of these products, ranged from 0 to 300. To account for the multifocality and intratumoral heterogeneity of PC, the final COX-2 expression score for each patient was calculated as the mean H-score obtained from the three corresponding cores from the primary tumor and, separately, from the three metastatic cores. COX-2 immunoreactivity in the study cohort was generally weak and heterogeneous, frequently limited to a few isolated tumor cells, whereas more extensive staining was observed only in a small subset of cases. As no externally validated H-score cut-off is available for COX-2 expression in node-positive prostate cancer, an exploratory, cohort-specific threshold of 25 was used as an operational criterion to distinguish absent or minimal focal staining from more substantial tumor-cell immunoreactivity. Cases with a mean H-score of ≤25 were classified as having low expression, whereas those with a mean H-score of >25 were classified as having high expression. Only COX-2 immunoreactivity in malignant epithelial cells was included in the H-score assessment; staining in inflammatory and stromal cells was not scored. IHC assessment of marker expression was independently conducted by two experienced genitourinary pathologists who were blinded to the clinicopathological data. Any discrepancies in scoring were resolved by consensus to establish the final H-score. No formal measure of interobserver agreement was calculated.

### 2.3. Statistical Analysis

The mean, standard deviation (SD), minimum (Min), maximum (Max), median (Me), lower quartile (Q1), and upper quartile (Q3) were calculated for quantitative variables. The empirical distribution of quantitative variables was examined for fit to a normal distribution using the Kolmogorov–Smirnov and Shapiro–Wilk tests. Spearman’s rank correlation coefficient was calculated to assess the association between monotonic relationships among variables. Differences in COX-2 expression levels between sample types were assessed using the Wilcoxon signed-rank test. For exploratory subgroup comparisons, expression levels were categorized as low (H-score ≤ 25) and high (H-score > 25) using the cohort-specific threshold described above. The Mann–Whitney U test was used to assess differences in quantitative parameters between two independent groups with different expression levels of the COX-2 marker, while Pearson’s chi-squared test was employed to evaluate the independence of two qualitative factors. Kaplan–Meier survival analysis was performed to assess the impact of COX-2 expression on 5-year overall survival, with comparisons between groups conducted using the log-rank test. A significance level of *p* < 0.05 was applied for all statistical tests. All statistical analyses were performed using Statistica v.13.3 (TIBCO Software Inc., Palo Alto, CA, USA).

## 3. Results

Positive COX-2 staining was observed in 75.3% (58/77) of primary prostate cancer specimens and in 63.6% (49/77) of corresponding LN metastases. In many samples, the expression was limited to scattered tumor cells.

No statistical difference was observed in COX-2 expression levels between primary prostate tumors and LN metastases (median H-score: 1.5 vs. 0.5; *p* = 0.099) (Table 1).

The general characteristics of the patients are presented in Table 2.

**Figure 1:**
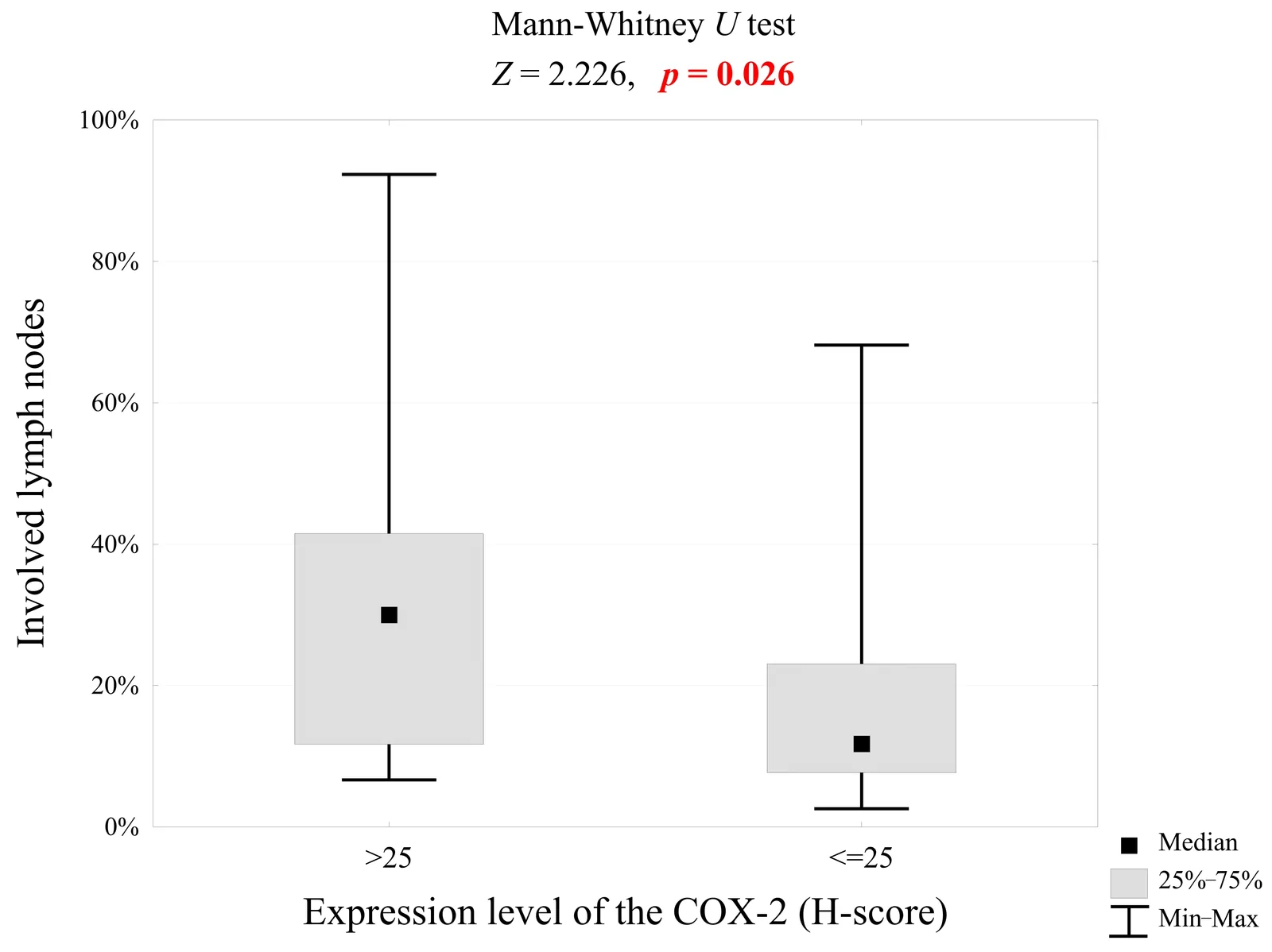
Percentage of involved lymph nodes stratified by COX-2 expression levels in the primary tumor (H-score).

Patients with high COX-2 expression in the primary tumor presented a significantly higher percentage of involved lymph nodes (median 30.0% vs. 11.8%; *p* = 0.026, Figure 1).

**Figure 2:**
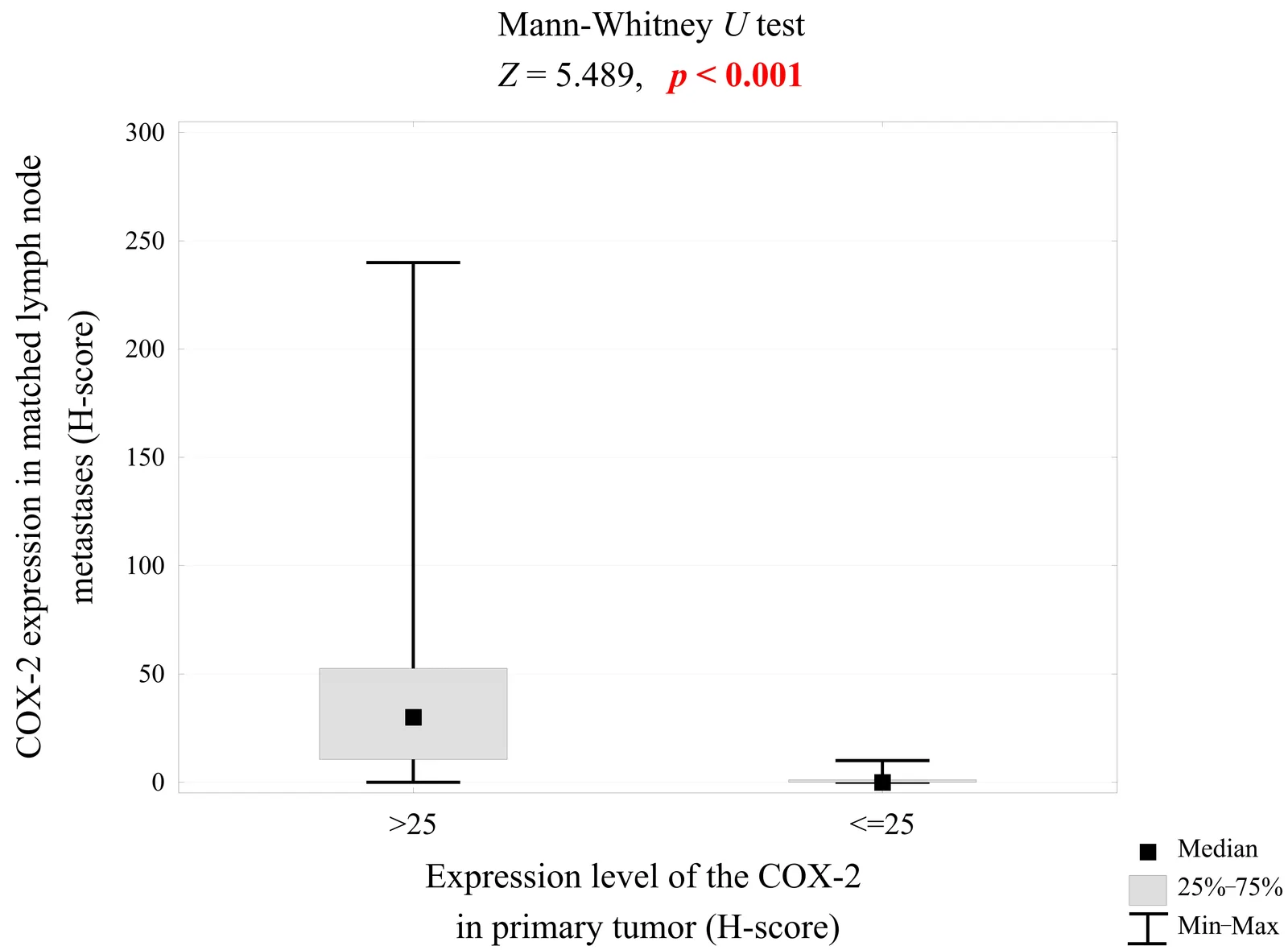
Comparison of COX-2 expression in lymph node metastases (H-score) according to COX-2 expression in the primary tumor (H-score).

Elevated COX-2 expression in primary tumor specimens was positively correlated with COX-2 expression in matched LN metastases (median H-score 30 vs. 0; *p* < 0.001, Figure 2).

**Figure 3:**
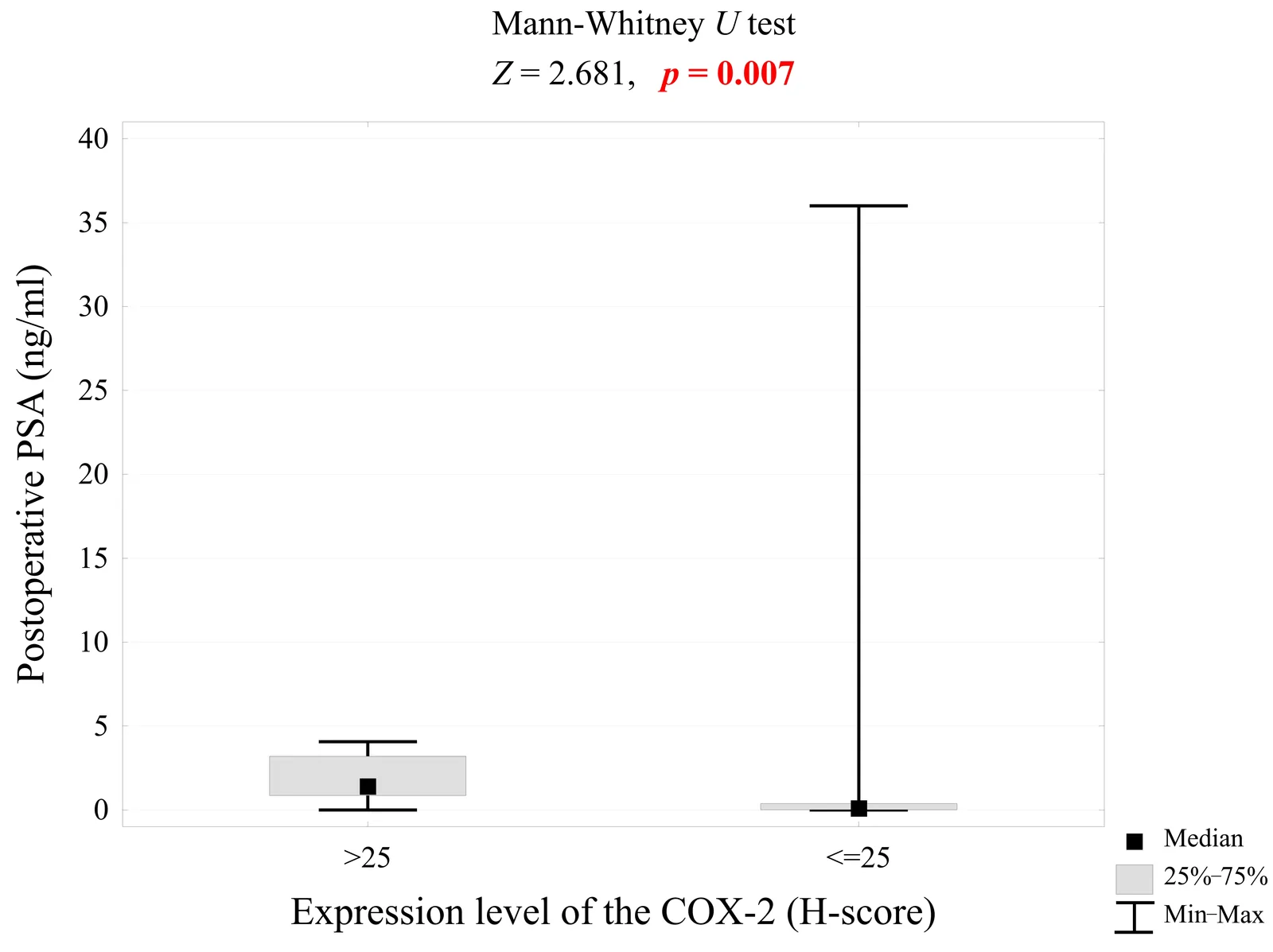
Comparison of the PSA level after surgery in relation to COX-2 expression in primary prostate tumors (H-score).

Notably, patients with high COX-2 expression in the primary tumor presented significantly higher postoperative PSA (1.98 vs. 0.10 ng/mL; *p* = 0.007, Figure 3).

**Figure 4:**
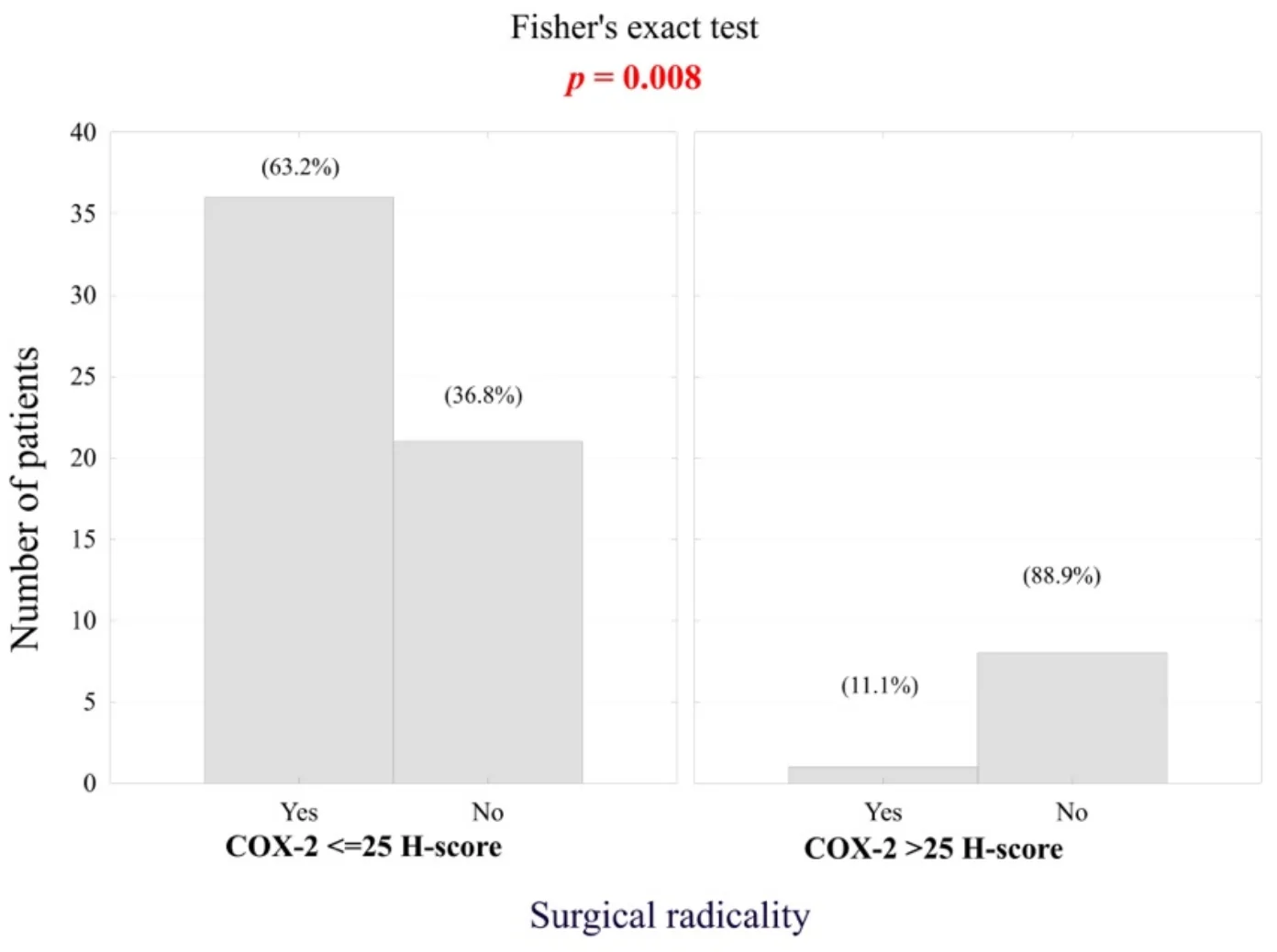
Comparison of percentage of radical procedures according to COX-2 expression in the primary tumor (H-score).

Surgical radicality was negatively associated with COX-2 expression level in the primary tumor (11.1% vs. 63.2%; *p* = 0.008, Figure 4).

**Figure 5:**
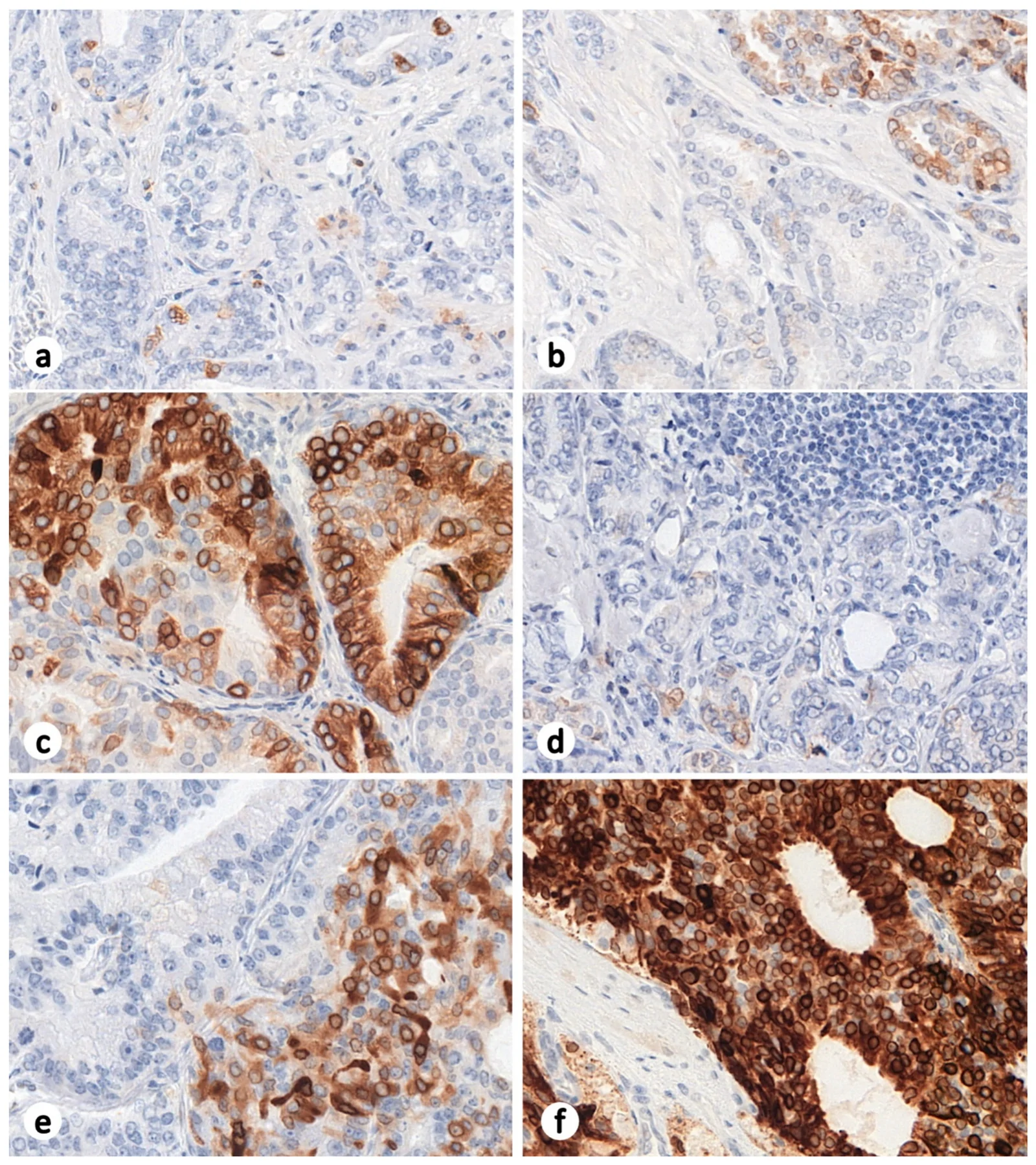
Representative examples of COX-2 expression in primary prostate cancer (**a**–**c**) and metastatic lymph nodes (**d**–**f**). (**a**): scattered positive cells, mean H-score = 1, low expression; (**b**): focal staining with weak-to-moderate intensity, mean H-score = 10, low expression; (**c**): strong immunoreactivity in the majority of cancer cells, mean H-score = 180, high expression; (**d**): few COX-2-positive tumor cells, mean H-score = 0.5, low expression; (**e**): patchy expression with moderate to strong staining intensity, mean H-score = 30, high expression; (**f**): diffuse strong positivity, mean H-score = 240, high expression. Magnification: ×200.

Representative examples of COX-2 immunohistochemical staining in primary prostate cancer and lymph node metastases are shown in Figure 5.

Mortality was significantly higher in the group with high expression than in the low-expression group (37.5% vs. 3.6%; *p* = 0.012).

**Figure 6:**
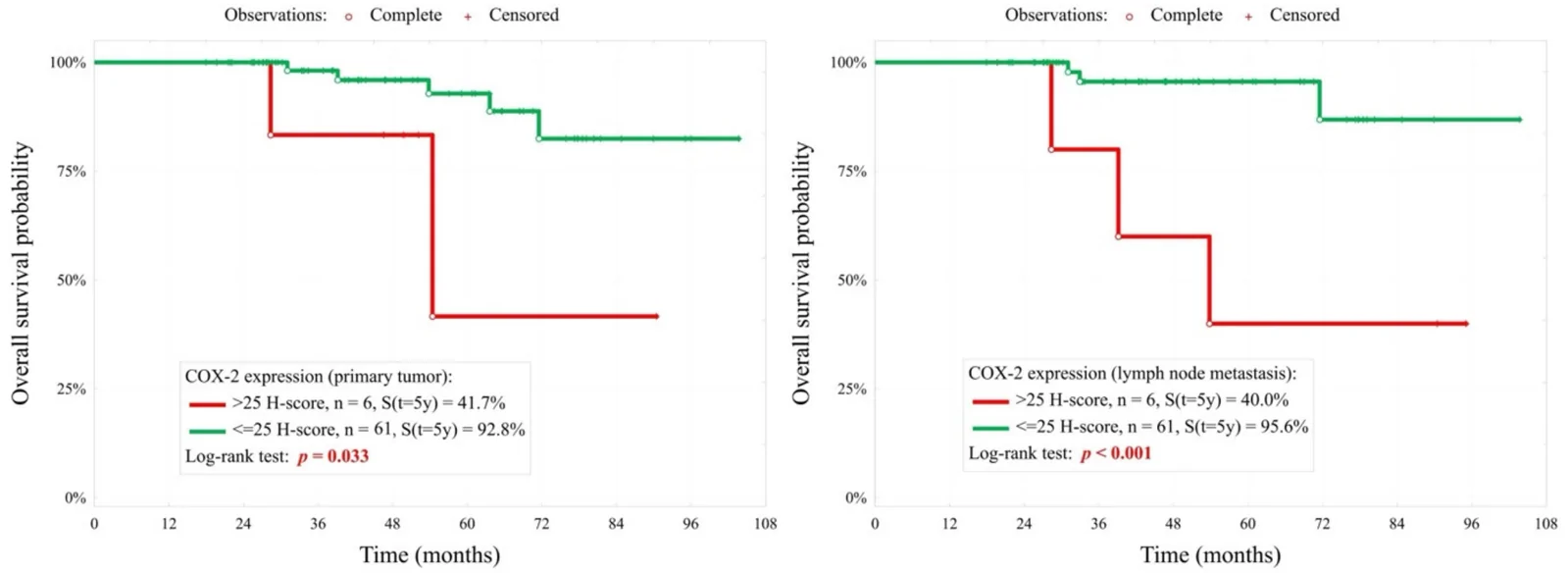
Kaplan–Meier survival curves of patients after radical prostatectomy in groups stratified according to COX-2 expression in primary tumors and lymph node (LN) metastases, 5-year survival values and log-rank test results.

Finally, 5-year survival was negatively associated with COX-2 expression in both primary and metastatic tumors (41.7% vs. 92.8%; *p* = 0.033 and 40.0% vs. 95.6%; *p* < 0.001, respectively, Figure 6).

## 4. Discussion

COX-2 is a biomarker that has been widely studied in the context of potential utility as a target for antitumor therapy [33]. Although results of ongoing clinical trials regarding prostate cancer treatment with celecoxib are inconclusive, the potential prognostic value of COX-2 expression is still promising [34,35,36]. Several studies have reported its association with worse outcomes in PC patients [20,21,22,23]. This is particularly important given that prostate cancer represents a significant clinical challenge for urooncologists, being the second most commonly diagnosed cancer among men worldwide. Its incidence is expected to increase further in the near future due to its strong correlation with aging [37]. Although PC is highly prevalent, the molecular and genetic mechanisms driving its progression remain insufficiently characterized. Additionally, 27–53% of all patients after RP or radiotherapy experience biochemical recurrence [38,39,40]. Therefore, additional prognostic tools in PC are required, as current clinical assessments rely predominantly on traditional parameters such as PSA levels, Gleason Grade Group, and clinical stage [41].

The aim of this study was to evaluate COX-2 expression in postoperative primary PC samples and in matched LN metastases. Although COX-2 expression in primary PC tissue has been studied previously, our analysis demonstrates its presence also in LN metastases [42,43,44]. However, we did not observe significant differences in COX-2 staining scores between primary tumors and matched lymph node samples. Notably, high COX-2 levels in PC specimens were associated with increased expression in matched LN metastases, suggesting a consistent expression pattern. In terms of primary tumor staining, we observed a rather heterogeneous expression of COX-2 in PC cells, which is in line with a previous report [25].

Our analysis revealed that patients with a high level of COX-2 staining in primary prostate cancer had a significantly higher percentage of affected lymph nodes than those with low expression (30.0% vs. 11.8%; *p* = 0.026). The percentage of metastatic lymph nodes—commonly referred to as lymph node density—is widely recognized as a reliable indicator of clinical recurrence. Its prognostic value has been confirmed in numerous studies, underscoring its crucial role in guiding postoperative management and risk stratification in PC patients [45,46]. Elevated lymph node density may be linked to increased neovascularisation in COX-2-positive tissues, as COX-2 expression seems to be associated not only with local inflammation but also with angiogenesis. In PC tissue, COX-2-positive areas presented higher microvessel density than regions with lower COX-2 expression [25]. This relationship has also been documented in other tumors like breast, colon, liver and endometrial cancers [47,48,49,50]. In colon cancer cells, COX-2 transfection led to increased vascular endothelial growth factor (VEGF) production, which could be inhibited by NS398, a selective COX-2 inhibitor [51]. Moreover, Di et al. showed a strong correlation between COX-2 and VEGF-C expression in prostate cancer, the latter being a key mediator of lymphangiogenesis [20]. These findings highlight that COX-2 contributes to angiogenesis not only indirectly through inflammation, but also directly via interaction with proangiogenic factors such as VEGF, COX-2–derived prostaglandins, and fibroblast growth factor-2 [52,53]. These molecular mechanisms may partially explain the association between COX-2 expression and increased lymph node density in this study.

Surgical radicality was less common in patients with increased COX-2 expression (11.1% vs. 63.2%; *p* = 0.008) and mean postoperative PSA levels (1.98 vs. 0.10 ng/mL; *p* = 0.007) were higher in this group. Postoperative PSA level is strongly associated with an increased risk of biochemical recurrence and overall mortality in patients following RP [54,55,56,57]. Persistent PSA after RP is significantly associated with adverse clinicopathological features and poorer oncological outcomes compared with patients with undetectable PSA levels [58]. Moreover, it has been established as an independent predictor of both clinical recurrence and cancer-specific mortality, as it is commonly present in primary disseminated disease [59]. Our findings are consistent with a previous study by Cohen et al., where COX-2 expression was identified as an independent predictor of PC progression—defined as a PSA level ≥ 0.4 ng/mL on two consecutive measurements—over a mean follow-up period of 96 months [22]. COX-2 immunostaining in that study demonstrated sensitivity, specificity, and overall accuracy exceeding 80% for predicting biochemical recurrence [22]. Unlike Cohen et al., who linked COX-2 to long-term progression (PSA recurrence), we observed its association with early surgical outcomes (non-radical procedures and persistent PSA).

Although we demonstrated a link between lymph node density, postoperative PSA and surgical radicality—all of which are associated with tumor aggressiveness—we did not observe any significant association with Gleason grade or the pathological stage of the primary tumor. However, the impact of COX-2 levels on tumor grade has been indirectly suggested by studies on epithelial neoplasms, where COX-2-mediated prostaglandin signalling resulted in inhibition of cell differentiation [18,19]. In PC, the effect of COX-2 on tumor grade remains ambiguous. While some studies reported a correlation with Gleason score and tumor stage [21,25], others found no consistent upregulation of COX-2 in PC or high-grade prostatic intraepithelial neoplasia, and no correlation with either Gleason score or T stage [60]. The role of COX-2 in prostate cancer differentiation thus remains to be fully elucidated.

This study supports previous findings indicating worse outcomes in PC patients with COX-2 upregulation. Overall mortality was higher in patients with high COX-2 expression in the primary tumor. Additionally, 5-year survival rates were lower in patients with high COX-2 levels in both primary and metastatic tumors. In a previous study, COX-2 upregulation was associated not only with worse biochemical progression-free survival, but also with lymphatic vessel density and lymphatic vessel invasion [20]. Therefore, poor survival observed in the high COX-2 expression group in the current study could be explained by pro-metastatic activity of COX-2, as supported by the increased lymph node density in this group. Similarly, in a study by Bin et al., patients with high COX-2 expression in PC specimens had significantly lower disease-free and overall survival rates compared with the low-expression group. Also, COX-2 level independently predicted patient survival [21].

Another tumor-promoting activity of COX-2 is related to its influence on matrix metalloproteinases (MMP-2 and MMP-9), which degrade components of the extracellular matrix, thereby reducing tumor cell adhesion and promoting invasion [61]. Also, COX-2 upregulates pro-urokinase plasminogen activator, a key enzyme involved in matrix degradation, further enhancing the metastatic potential of cancer cells [62]. Additionally, COX-2 may contribute to prostate cancer progression and metastasis through the induction of epithelial–mesenchymal transition (EMT), which is a critical process whereby epithelial cells lose their polarity and adhesion properties, acquiring mesenchymal features such as increased motility and invasiveness [63]. COX-2-derived prostaglandin E_2_ modulates key EMT transcription factors such as Snail, Twist, and ZEB1, leading to downregulation of epithelial markers (e.g., E-cadherin) and upregulation of mesenchymal markers (e.g., vimentin, N-cadherin). This shift in cellular phenotype facilitates tumor cell detachment, motility, invasiveness, and dissemination [63,64]. Although direct studies in prostate cancer are limited, similar mechanisms have been observed in other cancer types [65,66]. Taken together, previous studies suggest that COX-2 may be involved in multiple processes related to tumor progression, including angiogenesis, EMT, and tumor cell invasiveness. However, these mechanisms were not investigated in the present study, and our data do not allow us to distinguish intrinsic tumor-cell regulation of COX-2 from the potential influence of the local inflammatory microenvironment.

Potential limitations of our study include the retrospective design and single-center setting, which restrict the generalizability of the results. While such factors are common in exploratory research, the findings require cautious interpretation and highlight the importance of prospective validation. The TMA-based analysis of three cores and a single metastatic lymph node per patient may not fully capture intratumoral and inter-metastatic heterogeneity. Moreover, although the slides were independently reviewed by two pathologists, interobserver agreement was not formally quantified. Additionally, a comparative analysis of COX-2 expression in malignant versus non-malignant tissues (e.g., benign prostatic hyperplasia) could further enhance the study’s robustness. Future studies should also investigate the impact of COX-2 level on lymph node status in PC patients. Furthermore, inflammatory infiltrates were not systematically assessed; therefore, their potential influence on tumor-cell COX-2 expression cannot be excluded. This should be addressed in future studies. Moreover, the H-score threshold was exploratory and cohort-specific rather than externally validated; therefore, the reported subgroup associations may be sensitive to the selected cut-off and require confirmation in an independent cohort. Finally, the present study does not establish whether COX-2 provides prognostic information beyond established clinicopathological factors such as PSA, Gleason Grade Group, pathological stage, and lymph node burden. This should be evaluated in larger cohorts using appropriately powered multivariable analyses.

## 5. Conclusions

This exploratory paired-tissue study demonstrated COX-2 expression in primary prostate tumors and matched lymph node metastases, with a consistent expression pattern across both sites. Higher COX-2 expression was associated with adverse clinicopathological features and poorer overall survival in univariate analyses. However, the present findings do not establish that COX-2 is an independent prognostic factor or that it improves risk stratification beyond established clinicopathological variables. Larger, independent cohorts with appropriately powered multivariable analyses are required to determine its potential prognostic relevance.

## Figures and Tables

**Table 1:** COX-2 expression in prostatic tumors and LN metastases from 77 patients after radical prostatectomy.

	Prostate Cancer	Lymph Node Metastases	Test Result
Marker expression:			Z = 1.648 *p* = 0.099
Mean (SD)	8.4 (24.2)	8.3 (29.5)	
Me [Q1; Q3]	1.5 [1; 4]	0.5 [0; 2]	
Min–Max	0–180	0–240	
Level of expression, *n* (%)			c2 = 0.78 df = 2 *p* = 0.675
Low (H-score ≤ 25)	65 (84.4%)	69 (89.6%)	
High (>25 H-score)	12 (15.6%)	8 (10.4%)	

**Table 2:** Descriptive statistics of socio-demographic and clinical characteristics in groups of patients differing in the level of COX-2 expression (^a^—Mann–Whitney *U* test; ^b^—*t*-test; ^c^—chi-square test; ^d^—Fisher’s exact test).

Variable	COX-2 Expression in Prostate Cancer		*p*-Value
	High H-Score > 25 N = 12	Low H-Score ≤ 25 N = 65	
Body weight (kg), Me [Q1; Q3]	73 [71; 89]	88 [80; 95]	**0.029** ^a^
Height (cm), Me [Q1; Q3]	175 [169; 179]	176 [171; 180]	0.207 ^a^
BMI (kg/m^2^), mean (SD)	26.4 (4.6)	28.4 (3.5)	0.079 ^b^
Anesthesiologists Classification, n (%)			0.684 ^c^
ASA I	3 (25.0%)	8 (12.5%)	
ASA II	8 (66.7%)	47 (73.4%)	
ASA III	1 (8.3%)	8 (12.5%)	
ASA IV	0 (0.0%)	1 (1.6%)	
Karnofsky (score)	100 [90; 100]	100 [90; 100]	0.889 ^a^
ECOG			0.904 ^c^
0	8 (66.7%)	41 (64.0%)	
1	4 (33.3%)	22 (34.4%)	
2	0 (0.0%)	1 (1.6%)	
Preoperative PSA (ng/mL), Me [Q1; Q3]	26.1 [14.2; 36.6]	19.0 [9.0; 33.7]	0.389 ^a^
EAU risk group:			0.975 ^c^
1	0 (0.0%)	1 (1.6%)	
2	1 (8.3%)	6 (9.4%)	
3	6 (50.0%)	32 (50.0%)	
4	5 (41.7%)	25 (39.0%)	
Age (years), mean (SD)	63.8 (6.0)	64.9 (5.4)	0.518 ^b^
Hospitalization (days), Me [Q1; Q3]	6.5 [5; 8]	7 [6; 8]	0.448 ^a^
pT, n (%)			0.499 ^c^
pT2	0 (0.0%)	9 (13.8%)	
pT3a	1 (8.3%)	13 (20.0%)	
pT3b	11 (91.7%)	43 (66.2%)	
Gleason postoperative:			0.900 ^c^
3 + 3	0 (0.0%)	1 (1.5%)	
3 + 4	2 (16.8%)	9 (13.8%)	
4 + 3	1 (8.3%)	17 (26.2%)	
3 + 5	1 (8.3%)	3 (4.6%)	
4 + 4	1 (8.3%)	3 (4.6%)	
5 + 3	0 (0.0%)	2 (3.1%)	
4 + 5	6 (50.0%)	22 (33.8%)	
5 + 4	1 (8.3%)	7 (10.8%)	
5 + 5	0 (0.0%)	1 (1.5%)	
Postoperative GGG ISUP			0.721 ^c^
1	0 (0.0%)	1 (1.5%)	
2	2 (16.7%)	9 (13.8%)	
3	1 (8.3%)	17 (26.2%)	
4	2 (16.7%)	8 (12.3%)	
5	7 (58.3%)	30 (46.2%)	
ECE, n (%)	11 (91.7%)	55 (84.6%)	1.000 ^d^
Positive surgical margin, n (%)	8 (66.7%)	47 (72.3%)	0.734 ^d^
Involved lymph nodes (%), Me [Q1; Q3]	30.0 [11.7; 41.5]	11.8 [7.7; 23.1]	**0.026** ^a^
Extranodal extension, n (%)	4 (33.3%)	15 (23.1%)	0.476 ^d^
COX-2 in lymph node metastases (H-score), Me [Q1; Q3]	30 [10.5; 52.5]	0 [0; 1]	**<0.001** ^a^
NVI	n = 11 11 (100.0%)	n = 60 59 (98.3%)	1.000 ^d^
LVI	n = 11 10 (90.9%)	n = 61 48 (78.7%)	0.679 ^d^
Lymph nodes removed per patient, Me [Q1; Q3]	n = 12 13 [11; 20]	n = 65 20 [16; 25]	**0.010** ^b^
Positive lymph nodes per patient, Me [Q1; Q3]	n = 12 4 [2; 6]	n = 65 2 [1; 5]	0.319 ^b^
Postoperative PSA (ng/mL), Me [Q1; Q3]	n = 9 1.98 [0.86; 3.20]	n = 57 0.10 [0.02; 0.38]	**0.007** ^a^
Surgical radicality	n = 9 1 (11.1%)	n = 57 36 (63.2%)	**0.008** ^d^
Death (yes)	n = 8 3 (37.5%)	n = 56 2 (3.6%)	**0.012 ** ^d^

ECOG, Eastern Cooperative Oncology Group; EAU, European Association of Urology; ISUP GGG, International Society of Urological Pathology Gleason Grade Group; ECE, extracapsular extension; NVI, neurovascular invasion; LVI, lymphovascular invasion.

## Data Availability

The datasets used and/or analysed during the current study are available from the corresponding author on reasonable request.

## Abbreviations

The following abbreviations are used in this manuscript:

PSA	Prostate-specific antigen
PC	Prostate cancer
COX-2	Cyclooxygenase-2
PGs	Prostaglandins
COX-1	Cyclooxygenase-1
VEGF	Vascular endothelial growth factor
TGF-β	Transforming growth factor beta
NSAIDs	Non-steroidal anti-inflammatory drugs
5-FU	5-Fluorouracil
LN	Lymph node
RP	Radical prostatectomy
TMA	Tissue microarray
AJCC	American Joint Committee on Cancer
ISUP	International Society of Urological Pathology
EAU	European Association of Urology
IHC	Immunohistochemistry
H-score	Histological scoring system
SD	Standard deviation
Min	Minimum
Max	Maximum
Me	Median
Q1	Lower quartile
Q3	Upper quartile
MMP	Matrix metalloproteinase
NVI	Neurovascular invasion
LVI	Lymphovascular invasion
EMT	Epithelial–mesenchymal transition
